# The use of a novel reduction plate in transoral anterior C1-ring osteosynthesis for unstable atlas fractures

**DOI:** 10.3389/fsurg.2023.1072894

**Published:** 2023-05-03

**Authors:** Xiaobao Zou, Haozhi Yang, Chenfu Deng, Suochao Fu, Junlin Chen, Rencai Ma, Xiangyang Ma, Hong Xia

**Affiliations:** Department of Orthopedics, General Hospital of Southern Theatre Command of PLA, Guangzhou, China

**Keywords:** transoral approach, unstable atlas fracture, open reduction, internal fixation, atlantal plate

## Abstract

**Background:**

Transoral anterior C1-ring osteosynthesis has been reported as an effective treatment for unstable atlas fracture, which aims to preserve important C1–C2 motion. However, previous studies have shown that the anterior fixation plates used in this technique were not suitable for the anterior anatomy of the atlas and lacked an intraoperative reduction mechanism.

**Objective:**

This study aims to evaluate the clinical effects of a novel reduction plate used in transoral anterior C1-ring osteosynthesis for unstable atlas fractures.

**Methods:**

30 patients with unstable atlas fractures treated by this technique from June 2011 to June 2016 were included in this study. The patients' clinical data and radiographs were reviewed, and the reduction of the fracture, internal fixation placement, and bone fusion were assessed using pre- and postoperative images. The patients' neurological function, rotatory range of motion, and pain levels were evaluated clinically during follow-up.

**Results:**

All 30 surgeries were successfully performed, and the average follow-up duration was 23.5 ± 9.5 months (range 9–48 months). One patient suffered atlantoaxial instability during the follow-up and was treated with posterior atlantoaxial fusion. The remaining 29 patients had satisfactory clinical outcomes, with ideal fracture reduction, good screw and plate placement, well-preserved range of motion, neck pain alleviation and solid bone fusion. There were no vascular or neurological complications during the operation or follow-up.

**Conclusions:**

The use of this novel reduction plate in transoral anterior C1-ring osteosynthesis is a safe and effective surgical option in the treatment of unstable atlas fractures. This technique offers an immediate intraoperative reduction mechanism, which provides satisfactory fracture reduction, bone fusion, and preservation of C1–C2 motion.

## Introduction

Atlas fractures comprise a proportion of craniocervical injuries, acute cervical spine fractures, and all spine fractures, accounting for 25%, 2%–13%, and 1%–2%, respectively (1, 2). These fractures commonly occur at the weakest point of the atlas, which coincides with the attachment of the anterior and posterior arch in the lateral mass. Sköld's study has indicated that forehead injuries associated with extension generally cause posterior arch fractures, while axial compression due to an impact on the vertex is associated with anterior and posterior arch fractures (3).

Several classification systems for atlas fractures have been proposed. The most commonly used classifications in clinical research are the Jefferson, Landells and Van Peteghem, and Gehweiler classifications (4). The Gehweiler classification, which integrates categories from previous classifications, is considered more useful for clinical treatment (5). In prior literature, the presence or absence of injury to the transverse ligament (TAL) has been used to determine the stability of atlas fractures. Lee and Woodring's retrospective analysis of a large number of patients with atlas fractures suggests that single anterior arch fractures and posterior arch fractures without transverse ligament injury may be stable fractures, while other types are unstable fractures (6).

The optimal treatment for unstable atlas fractures remains a topic of debate, with no consensus on whether surgical or nonsurgical treatment is preferable. Nonsurgical treatments of unstable atlas fractures have been associated with poor reduction and high rates of nonunion, and neurological damage caused by instability of C0–C2 (7). Although posterior C1–C2 or C0–C2 fusion surgery can achieve satisfactory stability and bone fusion, it results in the loss of rotation of C1–C2 and flexion-extension of C0–C1 (8). In contrast, C1-ring osteosynthesis is an effective alternative to posterior C1–C2 or C0–C2 fusion for treating unstable atlas fractures while preserving important C1–C2 motion (2). Previous studies have reported on transoral anterior C1-ring osteosynthesis, but the anterior fixed plates used were not suitable for the anterior anatomy of the atlas and lacked an intraoperative reduction mechanism (9, 10).

**Figure 1:**
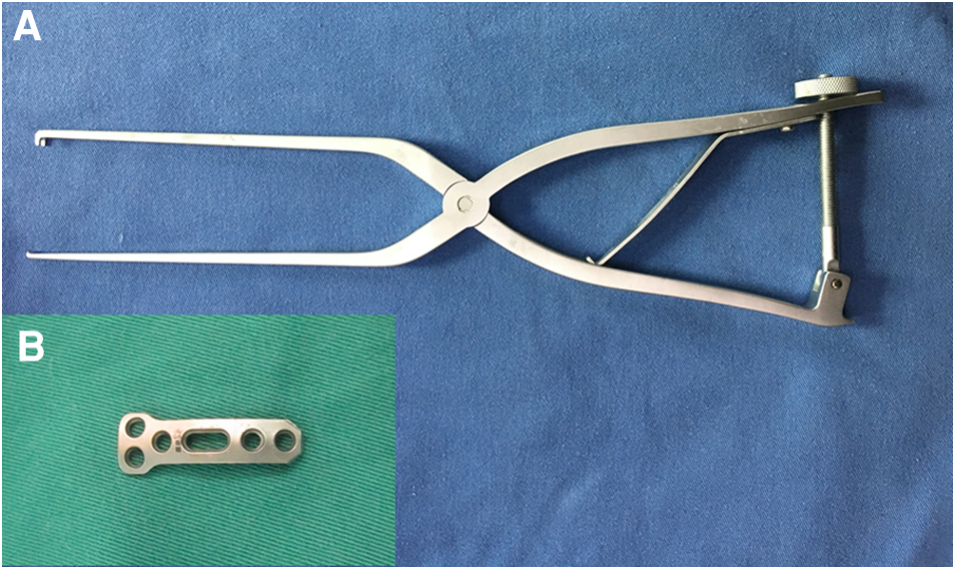
Novel instruments. (**A**) The reduction forceps. (**B**) The reduction plate.

This report presents a retrospective analysis of 30 patients with unstable atlas fracture treated using a novel reduction plate (Figure 1, Wego, Shangdong, China) for transoral anterior C1-ring osteosynthesis, and evaluates the preliminary clinical effects of this technique.

## Materials and methods

### Patients

This study was conducted in accordance with the Declaration of Helsinki, and approved by the Ethics Committee (IRB:20210033), with informed written consent obtained from each patient. From June 2011 to June 2016, a consecutive series of 30 patients with unstable atlas fractures were recruited and treated by transoral anterior C1-ring osteosynthesis using a novel reduction plate (Table 1). Prior to surgery, all patients underwent routine preoperative anteroposterior, open-mouth and lateral radiographs, computed tomography (CT), and magnetic resonance imaging (MRI).

**Table 1:** Clinical data of 30 patients.

Case	Gender	Age (years)	Injury cause	Fracture type	LMD (preop)	LMD (postop)	VAS (preop)	VAS (postop)	Bone fusion confirmed (month)	Follow-up (month)	Complications
1	F	40	Falling	III	6.1	0.0	5	1	3	12	No
2	M	42	MVA	III	5.5	0.0	8	1	3	36	No
3	M	66	MVA	III	6.1	1.7	7	1	6	36	No
4	M	24	Falling	III	6.5	2.5	8	2	6	48	No
5	M	52	MVA	III	12.3	2.5	6	1	9	24	No
6	F	26	Falling	III	7.2	0.0	7	1	3	24	No
7	F	42	Falling	III	4.3	0.0	8	2	3	24	No
8	M	28	MVA	III	6.5	0.0	6	1	3	12	No
9	M	65	Crushing	III	6.4	3.3	4	0	6	9	No
10	M	38	Falling	III	8.7	0.0	7	2	3	15	No
11	F	32	MVA	III	4.0	0.0	6	1	3	18	No
12	F	49	Falling	III	7.3	0.0	7	2	6	24	No
13	M	62	Falling	III	9.3	0.0	6	1	3	24	No
14	F	23	MVA	III	5.0	0.0	7	1	3	36	No
15	M	53	MVA	III	4.9	0.0	6	2	3	30	No
16	M	41	Crushing	III	6.4	0.0	8	1	3	24	No
17	F	47	MVA	III	6.6	2.0	7	2	6	12	Instability
18	M	58	MVA	III	4.8	0.0	8	2	3	24	No
19	M	47	Falling	III	8.1	0.0	6	1	3	36	No
20	M	45	MVA	III	7.2	1.5	8	1	6	24	No
21	F	43	Crushing	III	4.0	0.0	7	1	3	12	No
22	M	61	MVA	III	5.0	0.0	6	1	3	36	No
23	F	32	Crushing	III	6.2	0.0	7	2	3	12	No
24	F	53	Falling	III	4.0	2.5	8	3	9	18	No
25	F	66	MVA	III	4.7	0.0	7	2	3	30	No
26	M	51	MVA	III	5.2	1.9	8	2	6	21	No
27	M	41	Falling	III	12.4	3.6	6	1	9	24	No
28	M	41	Crushing	III	4.5	0.0	7	1	3	12	No
29	M	47	Falling	III	5.0	0.0	8	2	3	24	No
30	F	21	MVA	III	9.1	1.5	7	1	6	24	No
M ± SD					6.4 ± 2.2	0.8 ± 1.2	6.9 ± 1.0	1.4 ± 0.6	_ _		
t					15.739	36.546					
*P*					0.000*	0.000*					

M, male; F, female; MVA, motor verhicle accident; LMD, lateral mass displacement; VAS, visual analog scale.

* Paired-sample *t*-test.

### Surgical procedure

Preoperative preparation: Prior to surgery, patients were required to gargle six times daily with vinegar chlorhexidine, and underwent a professional dental cleaning. Intravenous ceftriaxone and ornidazole antibiotics were administered 30 min prior to the operation, and a nasogastric feeding tube was inserted.

**Figure 2:**
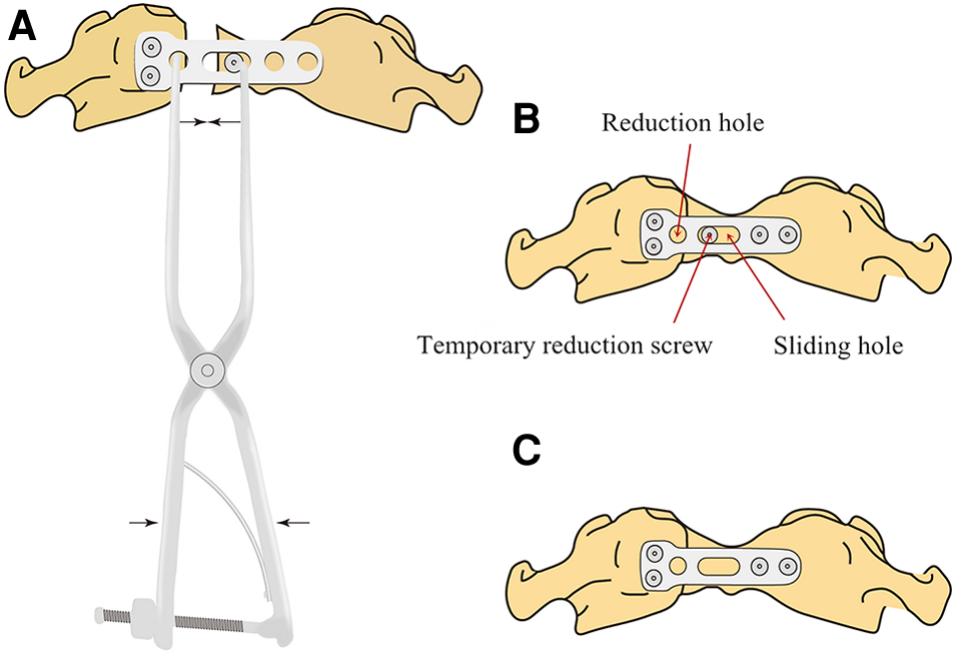
Reduction schematics of the novel plate for atlas fractures. (**A**) The forceps handles were closed to impart a compression force to reset the fracture after placement of the plate and temporary reduction screw. (**B**) Reduction of the fracture was achieved, and the plate was completely fixed. (**C**) The temporary reduction screw was removed.

Surgical technique: Under general anesthesia via nasal cannula, patients were positioned supine, and the oropharynx was cleaned and disinfected. The median posterior pharyngeal wall was then longitudinally incised about 3–4 cm to expose the anterior arch and lateral mass of the atlas. After verifying the location of the fracture, an appropriately sized plate was placed in front of the atlas. For a single fracture in the anterior arch, the wide end of the plate was fixed to the lateral mass near the fracture gap using two 18–26 mm screws. A temporary reduction screw was inserted into the anterior arch through the sliding hole of the plate. After C-arm fluoroscopy confirmed the position of the implanted device, a reduction forceps (Figure 1A) was installed between the reduction hole and temporary reduction screw. The forceps handles were then closed to apply compression force to close the fracture gap (Figure 2A). After confirming fracture reduction under direct vision, another two screws were placed in the atlas to fix the other end of the plate (Figure 2B), and the temporary reduction screw was removed (Figure 2C). For a double fracture in the anterior arch, a Crutchfield clamp was used to compress the lateral masses inwards to achieve fracture reduction (9), and then an appropriately sized plate was placed in front of the atlas to fix the fractures directly. C-arm fluoroscopic imaging was used to verify the location of the plate and screws, and the incision was closed in the muscular and mucosal layers.

### Postoperative management and follow-up

Postoperatively, patients had their tracheal cannula removed after 24–48 h, and nasogastric feeding tube removed after 7 days. Ultrasonic nebulisation and 0.02% chlorhexidine acetate gargling were administered 3–5 times daily for 7 days, and intravenous ceftriaxone and ornidazole antibiotics were given for 3 days. Cervical radiographs and CT scans were obtained 3 days after surgery to assess fracture reduction and the placement of fixation, as well as total lateral mass displacement (LMD). Patients were required to wear a rigid Philadelphia cervical collar for one month. Follow-up occurred at 3, 6, 9, and 12 months, and then once per year or as needed. Neck pain was assessed by visual analog scale (VAS), and the neurological status was also evaluated using the Japanese Orthopaedic Association (JOA) score (17-point system). Cervical radiographs and CT scans were performed at each follow-up to evaluate bone fusion of the fractures.

### Statistical analysis

The present study employed the Kolmogorov-Smirnov test to assess the normal distribution of the data, which were subsequently reported as mean and standard deviation. The statistical analysis of the data was performed using the paired-samples *t*-test and was conducted using SPSS 21.0 software (IBM, Armonk, NY, USA). A significance level of *p* < 0.05 was deemed appropriate to determine the statistical significance of the results.

## Results

### Characteristics of the study population

The study population consisted of 30 patients, comprising 18 men and 12 women with a mean age of 44.5 years (range 21–66 years). The causes of injury were falling (11 cases), motor vehicle accident (14 cases), and crushing (5 cases). All patients presented with neck pain and restricted motion of the cervical spine without neurological symptoms. Additionally, all patients had a JOA score of 17. In 9 cases, the fractures had failed to unite by using primary conservative treatment for 3 to 6 months, which included occiputocervicothoracic cast in 5 cases, rigid collar in 3 cases, and halo-vest in 1 case. The combined fractures of the anterior and posterior atlantal arches were found on CT images in all cases in this study, which were classified as type III fractures according to Gehweiler classification system (5). 9 patients had Dickman type I transverse atlantal ligament (TAL) injury (disruption of the midportion of the transverse was found on MRI), while Dickman type II TAL injury (fractures or bony avulsion at the attachment site of TAL presented on CT images) (11) was presented in 13 cases.

### Surgical results

All 30 surgeries were performed successfully without any neurovascular injury. The mean operative time was 78.3 ± 17.0 min (range 55–110 min), with an average intraoperative blood loss of 54.0 ± 22.2 ml (range 20–100 ml).

### Radiological results

**Figure 3:**
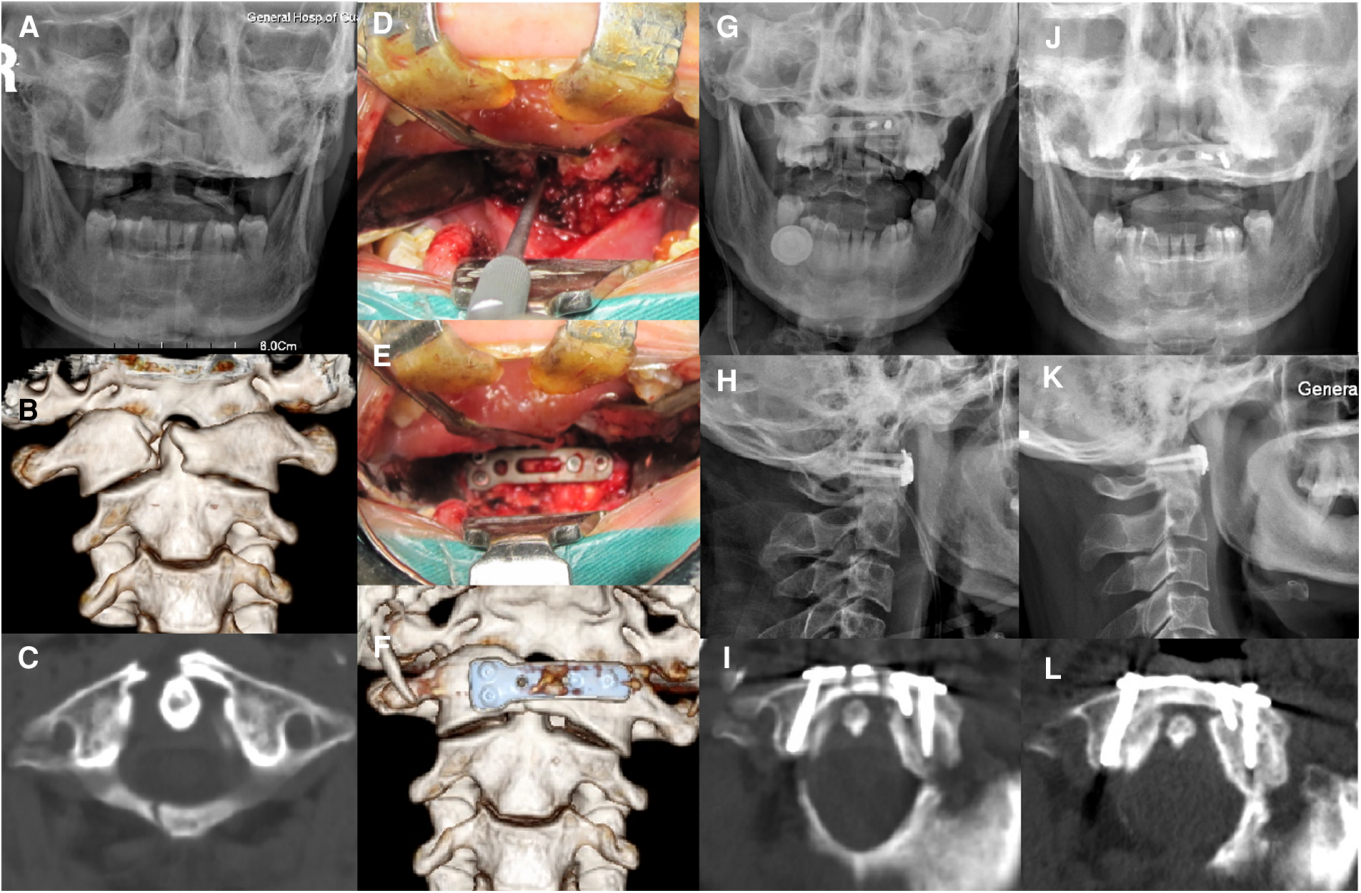
A 47-year-old female with combined fractures of the anterior and posterior atlantal arches was treated by transoral anterior C1-ring osteosynthesis using the novel reduction plate. (**A**) Preoperative open-mouth x-ray imaging showed displacement of the lateral masses. (**B,C**) The reconstructed images in the coronal and axial CT scan revealed fractures through the right side of the anterior and posterior arches of the atlas with displacement of the lateral mass. (**D,E**). Intraoperative photographs of an anterior arch fracture before and after fixation. (**F**) The reconstructed images after surgery showed optimal plate location. (**G,H**). Postoperative open-mouth and lateral x-ray imaging identified the relatively good C1–C2 alignment. (**I**) An axial CT image after surgery revealed reduction of the anterior arch fracture and adequate screw placement. (**J,K**) Open-mouth and lateral x-ray images at 6 months after surgery showed no loosening of the plate or screws. (**L**) An axial CT image at 6 months after surgery revealed solid bone fusion.

Postoperative CT scan revealed that plates and screws were well-placed in all cases (Figure 3), and the postoperative LMD (0.8 ± 1.2 mm, range 0.0–3.6 mm) significantly decreased compared to preoperative LMD (6.4 ± 2.2 mm, range 4.0–12.4 mm) (*p* < 0.01). None of the patients had screw or plate loosening or breakage after CT scans and plain radiographs during the follow-up period. One patient exhibited atlantoaxial instability (anterior atlanto-dental interval greater than 3 mm in flexion) during dynamic cervical radiograph 9 months after surgery and underwent posterior atlantoaxial fusion revision surgery. All other 29 cases had successful bone fusion after 3–9 months, with the patient who underwent revision surgery achieving bone fusion 6 months post revision surgery (Table 1). The postoperative cervical rotatory range of motion of the 29 patients was 48.9° ± 10.6° with a range of 35.8°–65.3°. All 29 patients had well-preserved range of motion.

### Clinical results

The 29 patients were followed up for a period ranging from 9 to 48 months, with an average of 23.5 ± 9.5 months, while the patient who underwent revised surgery was followed-up for 12 months. All patients maintained similar neurological functions to preoperative levels, with a JOA score of 17. The preoperative VAS scores (6.9 ± 1.0; range 4–8) were significantly reduced (1.4 ± 0.6; range 0–3; *p* < 0.01) after surgery. No complications of infection were observed as complications.

## Discussion

The atlas, also known as the first cervical vertebra, is a ring-shaped structure formed by the anterior and posterior arches and the two lateral masses without a vertebral body and spinous process. The regions where the anterior and posterior arches connect with the lateral masses are relatively thin and represent the weakest points of the atlantal ring. As a result of this unique anatomy, the atlas is most commonly fractured with two or more breaks in the ring structure (12). The stability of atlas fractures was traditionally determined by the structural integrity of the TAL (2). However, recent studies have shown that combined fractures of the anterior and posterior atlantal arches are unstable, regardless of the TAL involvement (6, 10, 13).

Patients with atlas fracture rarely present symptoms of neurological dysfunction, as there is an increase of the space available for the spinal cord after fractures of the atlantal ring, thereby preventing compression. While quadriplegia and hemiparesis may occur for a few minutes, these episodes generally fade away rapidly (14). Therefore, stabilization of fractures is the most important factor in the treatment of atlas fractures. While there is agreement on the treatment of stable atlas fractures, the optimal management of unstable atlas fractures remains controversial. Non-operative treatments such as skull traction and external immobilization using halo-vest or occiputocervicothoracic cast or rigid collar have been commonly suggested in the past (15). Although satisfactory outcomes could be obtained in most patients without associated neurologic deficits after nonoperative treatments, there is a high risk of nonunion (7). Mechanical instability and incongruence of the atlanto-occipital and the atlanto-axial joints may lead to arthrosis, persistent neck pain, and even neurologic injury. Dvorak et al. (16) reported that conservative treatments for atlas burst fractures failed to restore patients to their preoperative functional levels and suggested that nonoperative treatments were not optimal. Moreover, immobilization of the cervical spine for several months may lead to significant discomfort and other complications especially in elderly patients (17).

C1–C2 or C0–C2 fixation and fusion techniques, including C1–C2 transarticular screw fixation, C1–C2 screw-rod fixation, and occipitocervical plate-screw-rod fixation, are commonly used in surgical stabilization for unstable atlas fractures (18, 19, 20). These fixation techniques offer adequate biomechanical stability to achieve a high fusion rate (21, 22). However, they have certain limitations, such as loss of normal motion of the C1–C2 and C0–C1 joints and possible increased incidence of subaxial cervical spine degeneration (2).

In 2004, Ruf et al. (23) reported a transoral anterior C1-ring osteosynthesis technique that uses a lateral mass screw-rod construct to stabilize unstable atlas fractures while preserving C1–C2 motion, and obtained satisfactory clinical outcomes. Dickman proposed that the rupture of TAL results in permanent anterior instability of the C1–C2 joint (24). Alves et al. (5) recommended using C1-ring osteosynthesis to treat Gehweiler Type IIIb atlas fractures (combined injury of the anterior and posterior arch of the atlas with TAL damage) with a Dickman type II TAL injury. Kandziora et al. (4) suggested that Gehweiler Type IIIb atlas fractures with midsubstance ligamentous disruption (Dickman type I) or severely dislocated ligamentous bony avulsions (Dickman type II) of the TAL should be treated by C1–C2 fusion, while Gehweiler Type IIIb atlas fractures with moderately dislocated ligamentous bony avulsion (Dickman type II) of the TAL may be treated by C1-ring osteosynthesis only. However, recent biomechanical researches have suggested that under physiologic loads, solitary C1 fixation can provide adequate stabilization, and the well-preserved longitudinal ligaments have sufficient capacity to maintain the stability of the C1–C2 joint even with concomitant TAL injuries in atlas fractures (25, 26). A retrospective clinical study form Shatsky et al. (27) suggested that C1-ring osteosynthesis can be performed in the setting of incompetent TAL regardless of TAL injury type without resulting in C1–C2 instability. C1-ring osteosynthesis is currently considered a valid alternative to posterior C1–C2 or C0–C2 fusion for unstable atlas fractures, with or without TAL injury.

C1-ring osteosynthesis could be performed by either a transoral anterior or posterior approach (2, 9, 10, 27). Reduction of anterior arch fractures is critical, as the healing of anterior arch is essential to restore atlantoaxial stability. Although the transverse screw-rod fixation could be used for compression reduction in posterior C1-ring osteosynthesis, the compression force only directly acts on the tail of the screws, and the force transferred to the front end of the screws is insufficient, leading to poor reduction of anterior arch fractures. A transoral anterior approach provides direct access to the anterior arch of the atlas, enabling optimal closure of anterior arch fractures under direct vision.

Previous studies have reported satisfactory effects for unstable atlas fractures using transoral anterior C1-ring osteosynthesis. However, these instruments were not perfectly suited to the anterior anatomy of the atlas according to a lateral mass screw-rod construct used by Ruf et al. (23) and a reconstruction plate used by Ma et al. and Hu et al. (9, 10). Therefore, a novel C1 anterior plate with a reduction mechanism was developed by our team for transoral anterior C1-ring osteosynthesis in unstable atlas fracture that can preserve normal C1–C2 rotatory motion.

In this study, we evaluated the clinical efficacy and safety of a novel reduction plate instrumentation technique in the treatment of unstable atlas fractures. A total of 30 patients with unstable atlas fractures were included in the study, and clinical efficacy and safety were evaluated on follow-up. One patient in the study had C1–C2 instability, which was corrected by posterior C1–C2 fusion. The remaining 29 patients achieved a well-preserved range of motion and satisfactory bone fusion without signs of instability or complications. The failure case was attributed to a possible obscure damage of other ligaments. This novel reduction plate instrumentation unified the burst fracture by compression forces using a configured reduction forceps. Wound infection may be a matter of concern during the transoral approach. With proper preoperative preparation and postoperative care, the complication rate can be markedly reduced (28). No wound infection occurred in our case series.

### Limitations

There are several limitations in this study. Firstly, the sample size is relatively small, which may limit the generalizability of the findings. Further studies with larger sample sizes are necessary to fully evaluate the safety and efficacy of this technique. Additionally, as this is a retrospective study, it is subject to potential biases and limitations inherent in this type of study design. A prospective study with standardized outcome measures and controlled follow-up intervals may provide more robust results. Furthermore, the lack of a control group in this study limits the ability to comprehensively evaluate the effects of this technique.

## Conclusion

Transoral anterior C1-ring osteosynthesis using this novel reduction plate is a safe and effective surgical option to manage unstable atlas fractures. This technique can provide satisfactory reduction, optimal stabilization and bone fusion of fracture, and preserve important C1–C2 motion.

## Data Availability

The raw data supporting the conclusions of this article will be made available by the authors, without undue reservation.
